# Optimal Deployment of Sensor Nodes Based on Performance Surface of Underwater Acoustic Communication

**DOI:** 10.3390/s17102389

**Published:** 2017-10-20

**Authors:** Sunhyo Kim, Jee Woong Choi

**Affiliations:** Department of Marine Science and Convergence Engineering, Hanyang University, 55 Hanyangdaehak-ro, Sangnok-gu, Ansan, Gyeonggi-do 15588, Korea; sunhyo4485@hanyang.ac.kr

**Keywords:** underwater acoustic sensor network, performance surface, virtual force-particle swarm optimization, optimal deployment

## Abstract

The underwater acoustic sensor network (UWASN) is a system that exchanges data between numerous sensor nodes deployed in the sea. The UWASN uses an underwater acoustic communication technique to exchange data. Therefore, it is important to design a robust system that will function even in severely fluctuating underwater communication conditions, along with variations in the ocean environment. In this paper, a new algorithm to find the optimal deployment positions of underwater sensor nodes is proposed. The algorithm uses the communication performance surface, which is a map showing the underwater acoustic communication performance of a targeted area. A virtual force-particle swarm optimization algorithm is then used as an optimization technique to find the optimal deployment positions of the sensor nodes, using the performance surface information to estimate the communication radii of the sensor nodes in each generation. The algorithm is evaluated by comparing simulation results between two different seasons (summer and winter) for an area located off the eastern coast of Korea as the selected targeted area.

## 1. Introduction

The underwater acoustic sensor network (UWASN) has recently attracted considerable attention because of its potential for use in a variety of military and civilian applications, such as ocean environmental monitoring, ocean exploration, target detection, surveillance systems, and communication among underwater sensor nodes [1,2,3,4].

In the UWASN, a number of sensor nodes are deployed in the ocean to collect data, which must be successfully exchanged among adjacent sensor nodes. For this reason, each sensor node must be positioned to be able to perform collaborative communication tasks over a targeted area. The deployment efficiency of sensor nodes in the UWASN is evaluated in terms of their connectivity and the area covered by the limited number of sensor nodes in the targeted area, because underwater sensor devices are more expensive and more difficult to deploy at precise positions compared to sensor nodes on land [5].

Most attempts to find an optimal deployment scheme for sensor nodes have assumed that every sensor node has the same sensing range [6,7,8,9], which is reasonable in the case of terrestrial wireless communication systems. However, underwater communication performance is significantly influenced by temporal and spatial variations in the ocean environment [10,11]. Underwater acoustic communication presents a challenging problem in that the underwater acoustic communication channel is a time-varying multipath channel formed by multiple interactions of sound with the sea surface and bottom of the ocean, particularly in shallow water.

This multipath channel causes significant delay in spreading, often covering hundreds of symbols, thereby causing inter-symbol interference (ISI). This happens because the underwater sound propagates with relatively low sound speed (mean sound speed in water is around 1500 m/s, which is approximately 200,000 times lower than that of an electromagnetic wave) [12]. The ISI results in a significant degradation in communication performance [13]. In addition, a time-varying channel produces a short coherence time or a large Doppler spread resulting from the temporal and spatial variations in the sea surface [14]. Therefore, if all sensor nodes are deployed at equal distances from each other in the targeted area under the assumption that each sensor node has the same communication radius, performance degradation of the UWASN may occur. 

It is necessary to develop an optimal deployment algorithm for underwater sensor nodes, which must be able to reflect performance variations in the sensor network due to environmental fluctuations. One of the methods to improve the performance of the UWASN is to use array processing. Array processing techniques using an array of spatially separated receivers have been reported to decrease communication error rates by eliminating the ISI caused by multipath channels in the ocean and to have better communication performance than single receivers that only use the equalizer technique [15,16,17]. In this paper, a multi-channel combining technique using a vertical line array is applied to the UWASN. The performance of the communication system is then estimated over the targeted area, which is converted to the communication performance surface (PS). The PS is a projection of the geospatial information of the performance of a system. In general, the PS in a sonar system is used to provide insight into the relative performance for the detection range of the system, demonstrating the impact of the ocean environment on the system capability [18]. Here, a communication PS is predicted based on the communication radius estimated on each grid within the targeted area. On the basis of the estimated communication PS, the optimal deployment of sensor nodes is obtained using a hybrid optimization method combining the advantages of a virtual force algorithm [19,20] and a particle swarm optimization [21,22], called virtual force-particle swarm optimization (VFPSO) [23]. The goal of this paper is to achieve the maximum communication coverage maintaining their connectivity with the limited number of underwater sensor nodes, especially in shallow water areas.

The paper is organized as follows. Section 2 provides the descriptions of four sub-algorithms for optimal deployment of the sensor nodes: modeling of underwater acoustic channel impulse response; estimation of the communication performance; construction of the communication PS, and; optimal deployment of the sensor nodes. Section 3 presents the results of the optimal deployment positions for sensor nodes simulated for winter and summer seasons at a targeted area located off the east coast of Korea. Finally, Section 4 provides a summary and conclusion.

## 2. Algorithm for Optimal Deployment

The algorithm to obtain the optimal deployment scheme is composed of four sub-categories. First, underwater acoustic channel modeling is carried out using a ray-based acoustic model for the targeted area using the ocean environment database including bathymetry, sound speed profiles, and sediment types. Second, the communication performance is estimated using the array processing technique of a single-input multiple-output (SIMO) system. The signal received at each array channel is simulated via a convolution of the communication source signal with the simulated channel impulse response. Third, the PS for the targeted area is derived using the estimated communication performance. Lastly, the optimal deployment scheme for the sensor nodes is determined using the VFPSO method based on the estimated PS. 

### 2.1. Modeling of the Underwater Acoustic Channel Impulse Response

The underwater communication channel is characterized by multipaths caused by the acoustic interactions with the sea surface and bottom interfaces, as well as the water medium itself, which causes the ISI, and resulting in serious distortion of communication signals. The ISI makes demodulation of the communication signal complex and difficult. Therefore, it is necessary to determine the optimal deployment scheme based on the communication PS estimated via communication channel modeling. The model input parameters associated with the ocean environmental information, including sound speed profiles, bathymetry, and sediment properties, are needed for accurate underwater acoustic channel modeling. Here, the bathymetry in the targeted area is extracted from ETOPO1, which is a global relief model of Earth’s surface integrating land topography and ocean bathymetry supplied by NOAA (National Oceanic and Atmospheric Administration) [24,25], with a spatial resolution of ~100 m. The sound speed profiles are extracted from the GDEM (Generalized Digital Environment Model) with a 1/4° horizontal grid resolution and a monthly time resolution [26]. The values of sediment mean grain size are obtained by interpolating the mean grain sizes measured from surficial sediment samples taken directly from 14 positions within the targeted area. The mean grain size values of the surficial sediment were converted to the sediment sound speed, density, and attenuation coefficient using the empirical formula developed by Jackson and Richardson [27].

**Figure 1:**
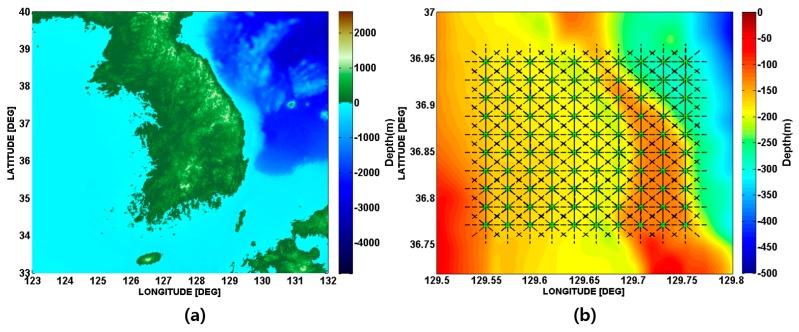
(**a**) Location of the targeted area and (**b**) 100 grid points in the targeted area. The lines in (**b**) indicate the directions of eight azimuthal angles at each point.

**Figure 2:**
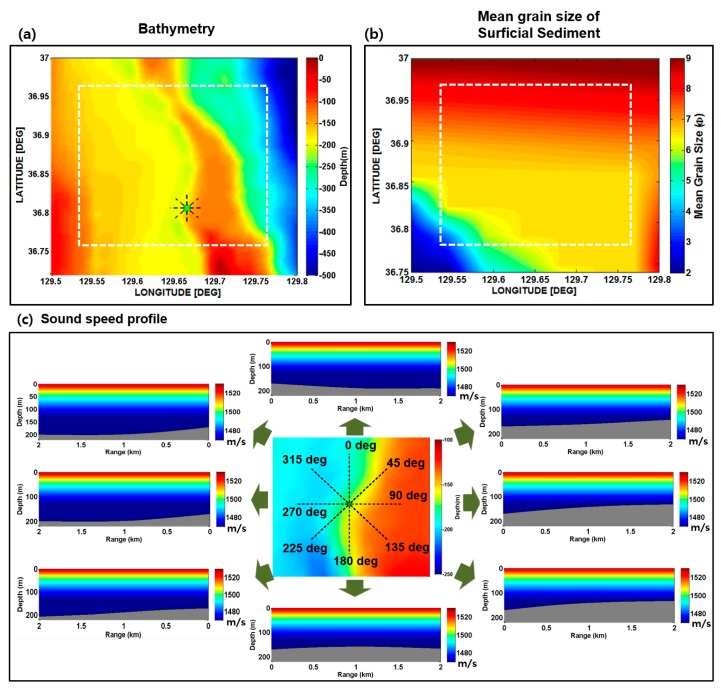
(**a**) Bathymetry map and (**b**) mean grain size distribution of surficial sediment of the targeted area. (**c**) Sound speed profiles and bathymetries for eight azimuthal angles of a grid point located at 36° 79′ N and 129° 68′ E, which is marked with a green circle in (**a**).

Figure 1a shows the targeted area selected in this paper. The size is 22 km × 22 km and its western boundary is ~10 km away from the eastern coast of Korea. The targeted area was divided into 100 grid points, for which environmental data were extracted from the database for eight azimuthal angles (Figure 1b). Figure 2a,b shows the interpolation maps of bathymetry and mean grain size (expressed by ϕ, where ϕ$=-\log_{2}\left(d\;/d_{0}\right)$, d is the grain diameter in millimeters, and $d_{0}$ is the reference length, equal to 1 mm) for the targeted area. Figure 2c shows examples of sound speed profiles and bathymetries for eight azimuthal angles of a grid point located at 36° 79′ N and 129° 68′ E. The deterministic channel impulse responses for the eight azimuthal directions are predicted using BELLHOP, which is a ray-based acoustic propagation model [28]. The BELLHOP model is a highly efficient and rapid acoustic ray tracing model suitable for higher frequency and range-dependent ocean environments.

**Figure 3:**
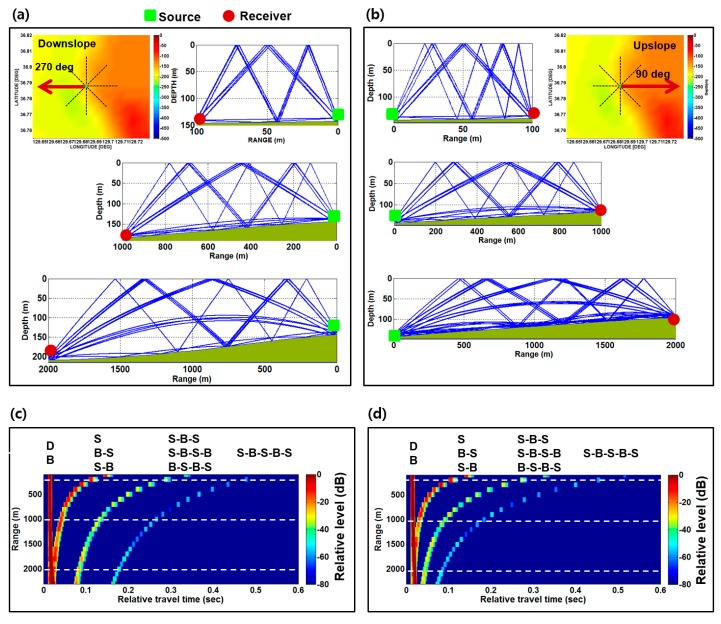
Eigenray tracing results for the azimuthal angles of (**a**) 90° and (**b**) 270°. Channel impulse responses as a function of arrival time for the azimuthal angles of (**c**) 90° and (**d**) 270°.

Figure 3a,b shows the eigenray tracing results for the azimuthal angles of 90° and 270°, respectively, at a grid point located at 36° 79′ N and 129° 68′ E. The eigenray tracing results for the source-receiver ranges of 100 m, 1 km, and 2 km are shown in the top, middle, and bottom plots, respectively. The source and receiver are assumed to be positioned 2 m above the bottom for both azimuthal angles. The main difference between the two different azimuthal angles is that the sound propagates into the up-slope direction for the azimuthal angle of 90° and the down-slope direction for the azimuthal angle of 270°. Figure 3c,d shows the channel impulse responses as a function of arrival time for the source-receiver ranges between 100 m and 2.5 km for the azimuthal angles of 90° and 270°, respectively. The first arrival includes direct (D) and bottom (B) paths. The second arrival includes the paths associated with single sea-surface bounce path, such as sea surface (S), bottom-surface (B-S), and surface-bottom (S-B) paths. The third and fourth arrivals correspond to the paths interacting with the sea surface twice and thrice, respectively. It is interesting to note that the delay spreads of the channel impulse responses for the down-slope direction are larger than those for the up-slope direction. This difference may eventually produce the different communication performance. The channel impulse responses simulated as a function of the source-receiver range for the eight azimuthal angles are convolved with an original communication sequence to obtain the received communication signals. This process is repeated for 100 grid points in the targeted area shown in Figure 1b. 

### 2.2. Estimate of the Communication Performance

Real-time communication with a high data transfer rate and low bit error rate (BER) is required for a robust UWASN system. There is a limit to using a single sensor node in the UWASN because underwater communication is severely affected by the time-varying multipaths in the ocean environment. Recently, in a number of studies on underwater acoustic communication, sensor array systems have been used to achieve the high data rate with low BER [15,16,17]. In general, as the element spacing and the number of sensors increase, communication performance becomes enhanced. However, the spatial diversity aggravates the spatial efficiency of the UWASN system. Here, it is assumed that each sensor node in the UWASN is a vertical line array composed of three hydrophone receivers with an element spacing of 1.5 m, which corresponds to 10 λ (where λ is the acoustic wavelength) based on a frequency of 10 kHz.

The communication sequence used for the communication performance simulation is a binary phase-shift keying (BPSK) sequence with a center frequency of 10 kHz and a bit rate of 1 kbps. To simulate the communication signals received after propagating through the multipath ocean channel, the BPSK communication sequence is convolved with the channel impulse responses simulated using the method described in Section 2.2, and then isotropic white Gaussian noise was added. 

**Figure 4:**
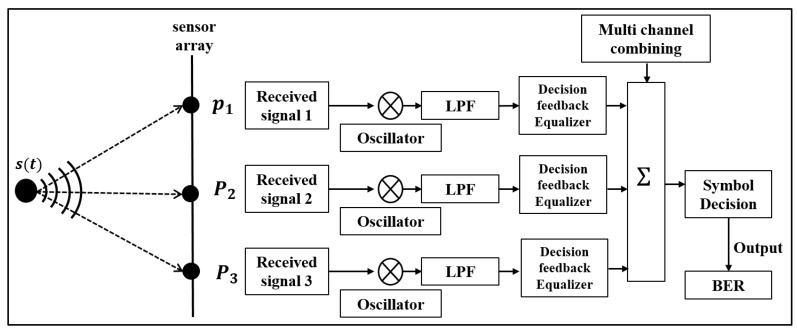
Block diagram of the single-input multiple-output (SIMO) communication receiver system.

Figure 4 shows a signal processing block diagram of the SIMO system used to decode the communication data. The communication data received by each sensor component are multiplied by $e^{-i\omega t}$ to recover the baseband waveform, where ω is the angular frequency. After that, the data were low-pass filtered and passed through an adaptive Decision feedback equalizer (DFE) [29] to compensate for the channel distortion by ISI. Recursive Least-Squares (RLS) algorithm was used to adaptively update the filter weights of the equalizer, and a forgetting factor was 0.995. The number of feedforward and feedback filter taps in the DFE is related to the delay time spread due to the multipaths in the underwater communication channel [10,17]. In this paper, the tap numbers covering the delay time spread sufficiently were used in the equalizer process. Then, they are summed to eliminate residual ISI and increase SNR (Signal-to-Noise Ratio) [30,31]. Finally, the communication performance is evaluated with a BER estimate.

### 2.3. Communication Performance Surface Algorithm

As described in the previous section, the process to estimate the communication performance is complex and time-consuming. If the process is repeated on candidate positions in the targeted area in the computation loop in order to search for optimal sensor positions, it will take too long to find the optimal solution. Here, the communication PS for the targeted area is constructed based on the BER performance estimated in Section 2.2, and then it is used for rapid computation of the search process to find the optimal positions. The communication PS represents the spatial distribution of the relative performance of the underwater communication system, which varies temporally and spatially in the ocean [18].

**Figure 5:**
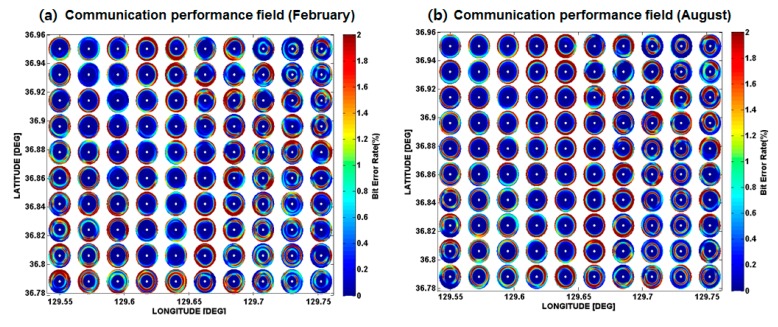
Communication bit error rate (BER) performance fields of 100 grid points obtained by interpolation of the BER estimate as a function of range for the eight azimuthal angles predicted using the mean sound speed profiles in (**a**) February and (**b**) August.

**Figure 6:**
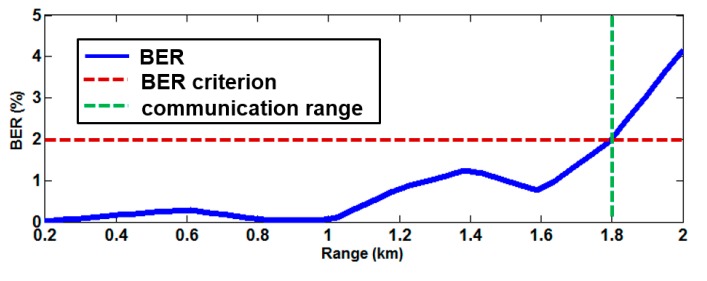
Example of the BER performance as a function of range. A BER of 2% is used as a criterion of tolerance for communication in this paper.

The BER estimates as a function of the source-receiver range for the eight azimuthal angles of each grid point were made to obtain the communication PS of the targeted area. Figure 5a,b show the interpolation of the BER performances for the eight azimuthal angles of each grid point predicted using the mean sound speed profiles in February and August, respectively. The difference between the sound speed profiles in the two seasons produces the difference of channel impulse response, which ultimately produces the difference in BER performance. Note that the BER may vary with the types of equalizer and receiver used in the UWASN. Here, a BER of 2% is chosen as a criterion of tolerance for communication, and the range corresponding to this criterion is defined as a communication range. Figure 6 shows an example of the BER performance estimated as a function of range. In this case, the communication range is determined to be 1.8 km.

**Figure 7:**
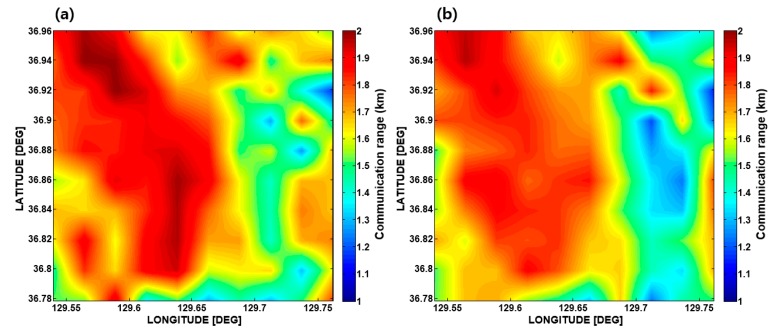
Communication performance surface (PS) simulated for the targeted area in (**a**) February and (**b**) August.

Now, the communication ranges for the eight azimuthal angles are averaged to represent the communication radius at the grid point. The minimum or maximum communication range can also be chosen optionally by a user as a representation of the communication radius at the grid point. The communication radii estimated for every grid point are interpolated to create the communication PS of the targeted area. Figure 7 shows an example of the communication PS simulated using a three-channel vertical array for the two different seasons ((a) in February and (b) in August). In our simulation case, the overall communication radii in February are longer than those in August, which means that the communication environment in February is better than that in August.

### 2.4. Optimal Deployment Algorithm for the Sensor Nodes 

In general, the performance of the algorithm for optimal deployment of the sensor nodes is evaluated in terms of maximizing the communication coverage rate and maintaining connectivity among the sensor nodes with a given number of nodes in the targeted area. Here, a hybrid method combining the advantages of the virtual force algorithm (VFA) [19,20] and the particle swarm optimization (PSO) [21,22] is used as a search algorithm, which is referred to as the VFPSO [23]. 

The VFA is a self-organizing search algorithm to determine the optimal distances among sensor nodes, which attempts to maximize the communication coverage using a combination of attractive and repulsive forces. As an initial step, the sensors are randomly placed in the targeted area. The distances between one sensor node and its neighboring nodes are compared to a communication threshold. In this work, the communication threshold was defined to be the smaller of the communication radii at two sensor node positions for two-way communication. The communication radius at a certain node position can be extracted from the communication PS map. If the distance is smaller than the threshold, a repulsive force arises between the two sensor nodes. In contrast, if the distance is too far apart, an attractive force arises. This process is performed for all neighboring nodes based on one sensor node, and a weighted sum is calculated to obtain the total force exerted on the node. This is repeated for each node. As the iteration of the algorithm continues, all sensors nodes are positioned, maintaining optimal distances between the sensor nodes.

The PSO is a stochastic search algorithm that mimics social behavior of animals moving in flocks in order to find optimal positions. It has been widely used because it is an efficient optimization algorithm for solving dynamic optimization problems having fast convergence and robustness [21,22]. The PSO uses particles, which correspond to the individual sensor nodes in this study, and the set of all sensor nodes is referred to as the swarm in the PSO algorithm. After being initialized with random positions and velocities, the particles estimate their communication radii at their positions and memorize this information. At each generation, particles evaluate the best positions by comparison with the positions achieved at previous generations. The velocity of each particle is adjusted based on the experiences of the particle and its companions. The position in the next generation is then updated by the sum of the present position and the adjusted velocity [23].

**Figure 8:**
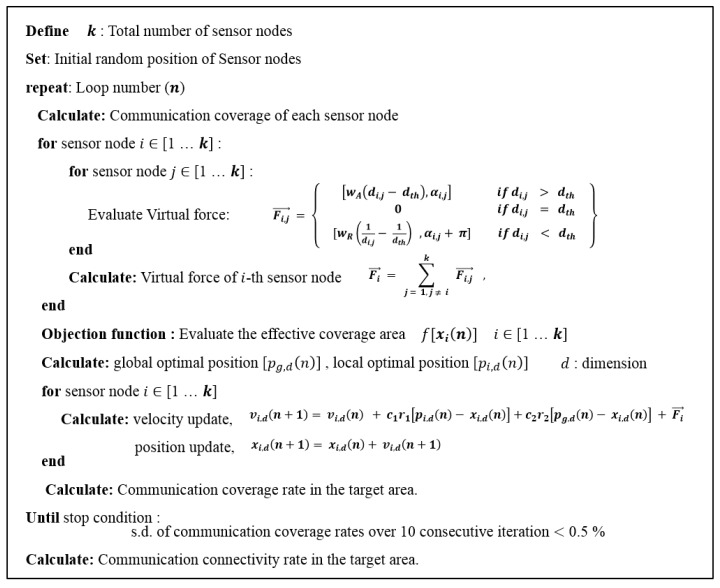
Pseudocode of the virtual force-particle swarm optimization (VFPSO) algorithm for the search of the optimal deployment positions for the sensor nodes.

**Figure 9:**
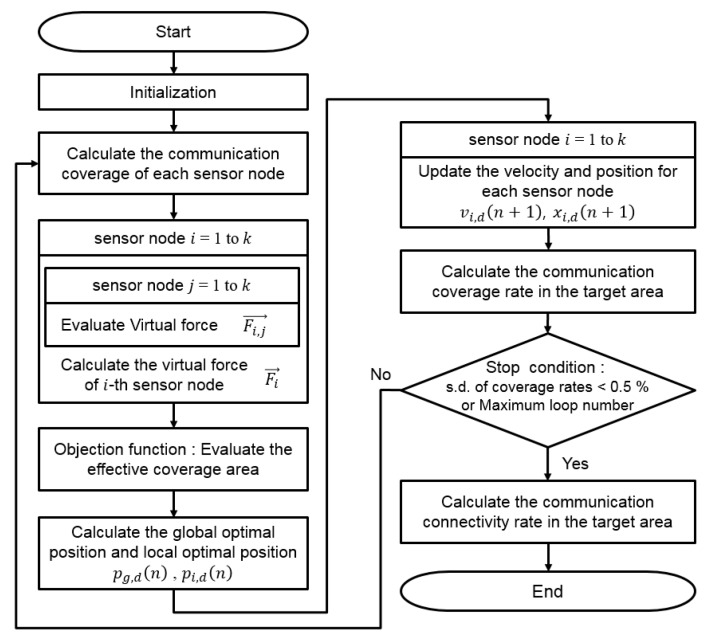
Flow chart of the VFPSO algorithm for the search of the optimal deployment positions for the sensor nodes.

**Figure 10:**
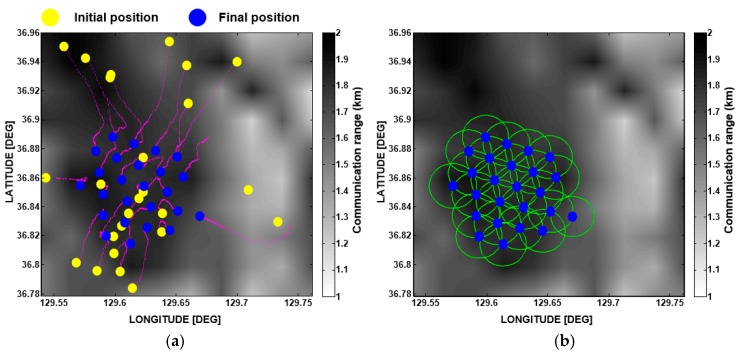
(**a**) Example showing the convergence progress of the sensor nodes. (**b**) Sensor positions after optimal deployment (blue dots). Green circles indicate the communication radii of the sensor nodes.

The VFA has outstanding performance in adjusting the distance among the sensor nodes. However, there may be a limit because the strong attractive and repulsive forces among the sensor nodes may hinder the nodes from settling at optimal positions. In contrast, the PSO has the ability to find a global optimal solution. However, it does not have the ability to adjust the distances among the sensor nodes, which hinders the maximization of the communication coverage rate. Here, the VFPSO algorithm is applied to find the optimal positions of the sensor nodes in the UWASN, which is expressed by [23].

(1) $$\vec{F_{i}}\;=\overset{k}{\underset{j\;=\;1,\;j\;\neq \;i}{\sum }}\vec{F_{i,\;j}}\;$$

(2) $$\vec{F_{i,\;j}}=\;\left\{\;\begin{array}{c} \left[w_{A}\left(d_{i,\;j}-\;d_{th}\right),\;\alpha_{i,\;j}\right]\;if\;d_{i,\;j}\;>\;d_{th}\\ \;0\;if\;d_{i,\;j}\;=\;d_{th}\\ \left[w_{R\;}\left(\frac{1}{d_{i,\;j}}-\;\frac{1}{d_{th}}\right)\;,\;\alpha_{i,\;j}+\;\pi \;\right]\;if\;d_{i,\;j}\;<\;d_{th} \end{array}\;\right\}\;$$

(3) $$\begin{array}{cc} v_{i,\;d}\left(n+1\right) & =v_{i,\;d}\left(n\right)+\;c_{1}r_{1}\left[p_{i,\;d}\left(n\right)-\;x_{i,\;d}\left(n\right)\right]\\ & \;+c_{2}r_{2}\left[p_{g,\;d}\left(n\right)-\;x_{i,\;d}\left(n\right)\right]+\;\vec{F_{i}}\; \end{array}$$

(4) $$x_{i,\;d}\left(n+1\right)=\;x_{i,\;d}\left(n\right)+\;v_{i,\;d}\left(n+1\right),\;$$

where $\vec{F_{i,\;j}}$ is the resultant force of the attractive force and repulsive force between the i-th and j-th sensor nodes. $\vec{F_{i}}$ is the total sum of the forces between the i-th sensor node and others. $d_{i,\;j}$ is the Euclidean distance between the i-th and j-th sensor nodes, and $d_{th}$ is the communication threshold, which was defined as the smaller value of the communication radii for the i-th and j-th sensor nodes as mentioned earlier. $\alpha_{i,\;j}$ indicates the direction from the i-th to j-th sensor nodes. $w_{A}$ and $w_{R}$ are the weighting values for the attractive and repulsive forces, respectively. $x_{i,\;d}\left(n\right)$ and $v_{i,\;d}\left(n\right)$ represent the position and velocity of the i-th sensor node in the *d*-th dimension at iteration *n*, respectively. $p_{i,d}\left(n\right)$ is the optimal position of the i-th sensor node until iteration n, and $p_{g,\;d}\left(n\right)$ is the optimal position obtained from the experiences of all sensor nodes until iteration n, which is called the global optimal position. The constants $c_{1}$ and $c_{2}$ are acceleration weight constants, and $r_{1}$ and $r_{2}$ are random numbers between 0 and 1. In this study, the effective communication coverage area was used as an objection function. The pseudocode for the VFPSO used to find the optimal positions for the sensor nodes is illustrated in Figure 8 and its flow chart is shown in Figure 9. The loop finishes when the communication coverage rate sufficiently converges and its standard deviation (s.d.) over 10 consecutive iterations becomes less than 0.5%. Figure 10 illustrates an example showing the convergence progress of the sensor nodes. Through iteration of the VFPSO simulation, the sensor nodes randomly distributed in the targeted area at the initial stage moved toward the optimal positions where network connectivity was well maintained.

## 3. Simulation Results

The optimal deployment performance of the sensor nodes based on the communication PS was simulated for two different seasons, winter (February) and summer (August), in the targeted area located off the eastern coast of Korea. Optimal deployment in our simulation means the sensor deployment having a maximized communication coverage while maintaining connectivity of the sensor nodes. Here, the communication coverage rate is defined as the ratio of the area of communication coverage by the sensor nodes to that of the targeted area. 

**Figure 11:**
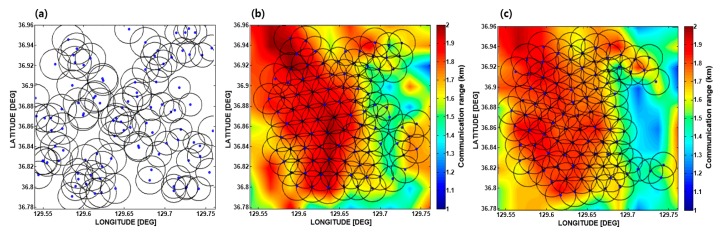
Examples of the optimal deployment simulation using 100 sensor nodes. (**a**) Randomly deployed initial positions. (**b**,**c**) are the optimal deployment results in February and August, respectively.

Figure 11 shows examples of the simulation results of optimal deployment for the two different seasons. The parameters used in the simulation are given in Table 1. The parameters belong in four sub-categories: environmental parameters; channel modelling parameters; communication parameters, and; optimal deployment parameters. For the simulation, 100 sensor nodes were used. As an initial step, 100 sensor nodes were randomly distributed in the targeted area, as shown in Figure 11a. The VFPSO search algorithm was then applied. The deployment results for February and August are displayed on their communication PS, which are illustrated in Figure 11b,c, respectively. The communication coverage rate for February was estimated to be 85.2%, which is higher than that for August (estimated to be 80.6%). This result is reasonable because the ocean condition of the targeted area in February provides a better underwater communication environment than that in August, as mentioned in Section 2.3.

**Figure 12:**
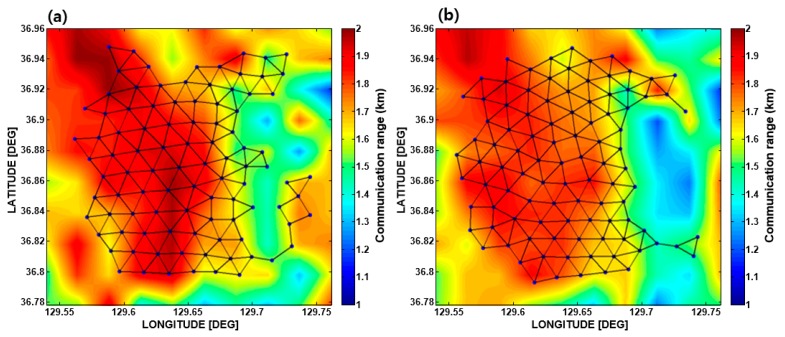
Network connections of the sensor nodes after optimal deployment in (**a**) February and (**b**) August.

Another important point to be examined is the connectivity among the sensor nodes, which is crucial for effective data communication of the UWASN system. The connectivity among the sensor nodes was estimated for the final deployment scheme to verify the performance of the VFPSO algorithm. The distance between each sensor node and its neighboring sensor nodes was compared to the communication threshold defined in Section 2.4, and the results for the two seasons are shown in Figure 12. The solid lines between sensor nodes indicate that effective two-way communication is possible. For both cases, 100% connectivity was achieved. Consequentially, the sensor nodes were positioned within the high communication performance area to achieve the maximal communication coverage rate, with intervals varying between 1.5 and 2.0 km for both seasons.

## 4. Summary and Conclusions

A new method for the optimal deployment of underwater sensor nodes, which can be applied to the UWASN, has been proposed in this paper. The algorithm includes four sub-algorithms, which are a routine for underwater acoustic channel modeling in temporally and spatially varying ocean environments, a routine for estimating the communication performance using the simulated underwater acoustic channels, a routine for constructing the communication PS, and a routine for searching for the optimal deployment of the sensor nodes on the PS. Most previous studies for the optimal deployment of sensor nodes have been performed under the assumption that the sensing range of every sensor node is the same without consideration of the spatial and temporal variations of the ocean environment. In contrast, the proposed method is an algorithm finding the optimal positions of sensor nodes using the communication radii extracted from the communication PS, which is the main difference from the previous algorithms. Moreover, it uses the VFPSO algorithm, which is a hybrid optimization method combining the advantages of the VFA and the PSO. Therefore, the sensor nodes are not placed in equidistant intervals. The optimal deployment based on the PS has been simulated for two different seasons for an area located off the eastern coast of Korea as the selected targeted area. As a result, the communication coverage rate in winter (February) was somewhat better than that in summer (August), implying that the optimal deployment scheme may vary with variations of the ocean environment. The algorithm does not propose the optimal number of sensor node, but it finds the optimal positions of sensor nodes for a given number of sensor nodes.

In this paper, it was assumed that the communication channel was time invariant. If the system is used for a short time period using portable sensors such as sonobuoy-type devices, the ocean parameters corresponding to that time should be used as input parameters. However, if it is for the long-term ocean surveillance monitoring for a certain area, it is highly recommended to use the ocean environmental parameters producing the worst communication radii to obtain the robust deployment scheme and, in this case, the sensor nodes would be closer to each other. Also, it was assumed that the BPSK signal with a center frequency of 10 kHz was transmitted as a communication sequence and each sensor node was composed of a three-receiver array with an element spacing of 1.5 m. Other communication sequences [such as OFDM (Orthogonal Frequency Division Multiplexing)] or a different type of receiver system, which might be more robust to (frequency/time-selective)] fading channels, could provide better communication performance and produce a different deployment scheme; however, these are beyond the scope of this paper. Nevertheless, our algorithm can be applied to other types of communication sequences and receivers. 

In this paper, it was assumed that all sensor nodes were positioned near the seafloor. The depth change of the sensor nodes may yield a better communication performance, and in addition the combinations of different types of sensors, including AUV (Autonomous Underwater Vehicle), may improve the performance. The algorithm suggested herein can be extended to these cases, which will be our future work. 

## Figures and Tables

**Table 1:** The parameters used in the optimal deployment simulation.

**Environmental Parameters**	**Value**	**Channel Modeling Parameters**	**Value**
Month	2, 8	Frequency	10 kHz
Longitude direction distance	22 km	Source level	140 dB
Latitude direction distance	22 km	Source depth	2 m above the bottom
Wind speed	10 m/s	Receiver depth (Three vertical receiver array)	0.5~3.5 m above the bottom
Azimuth angle interval	45°		
Grid points	100	Element spacing	1.5 m (10 λ)
**Communication Parameters**	**Value**	**Optimal Deployment Parameters**	**Value**
Symbol number	3500	Loop number	50
Symbol rate	1000 sps	Sensor node number	100
Pulse shaping	Root Raised Cosine filter	Weight value of attractive force	0.01
Equalizer	Adaptive DFE(RLS)	Weight value of repulsive force	0.5
BER criterion	2%	Acceleration weight	1
